# Effects of Infrared Radiation on Eggplant (*Solanum melongena* L.) Greenhouse Cultivation and Fruits’ Phenolic Profile

**DOI:** 10.3390/foods8120630

**Published:** 2019-12-02

**Authors:** Vassilia J. Sinanoglou, Angeliki Kavga, Irini F. Strati, Georgios Sotiroudis, Dimitra Lantzouraki, Panagiotis Zoumpoulakis

**Affiliations:** 1 Laboratory of Chemistry, Analysis & Design of Food Processes, Department of Food Science and Technology, University of West Attica, Ag. Spyridonos, 12243 Egaleo, Greece; estrati@uniwa.gr; 2 Department of Agricultural Science, University of Patras, University Campus, 26504 Rio Patra, Greece; akavga@upatras.gr; 3 Institute of Chemical Biology, National Hellenic Research Foundation, 48 Vas. Constantinou Ave., 11635 Athens, Greece; gsotir@eie.gr (G.S.); dlantzouraki@gmail.com (D.L.)

**Keywords:** infrared heating greenhouse, eggplant, antioxidant activity, radical scavenging activity, total phenolic content, LC/MS, phenolic profile

## Abstract

The implementation of Infrared (IR) radiation in heated greenhouses possesses the advantage of high directional control and focused compensation of energy losses, appropriate for creating local microclimate conditions in highly energy-consuming systems, such as greenhouses. Moreover, it can efficiently maintain favorable environmental conditions at the plant canopy. The present study studies the application of Infrared (IR) heating in an experimental greenhouse with eggplant (*Solanum melongena* L.) cultivation. The experimental results are presented from a full cultivation period inside two identical, small scale experimental greenhouses, with IR and forced air heating system, respectively. The effects of IR heating over plant growth parameters, including the yield of the fruits as well as the total phenolic content and the antioxidant profile of eggplants fruits’ extracts are measured and discussed. The results indicate a greater uniformity production in the IR heating greenhouse in terms of antioxidant and radical scavenging activities, as well as the total phenolic content. Moreover, the phenolic profile of eggplant fruits from both greenhouses revealed the existence of numerous bioactive compounds, some of which were only characteristic of the eggplant fruits from IR heated greenhouses.

## 1. Introduction

Greenhouses, as intensive cultivation structures, are aiming towards maximum crop production. A complex combination of microclimate factors, including heating and cooling, lighting, and ventilation, have to be controlled and subsequently optimized in order to achieve this goal [1].

In the temperate Mediterranean region, where illumination is adequate, a greenhouse is heated to control the internal air temperature. Greenhouse heating is the most energy-consuming operation, significantly increasing the production cost [2]. Heating is achieved by air heaters or by circulation of hot water in piping systems or a combination of the above systems in conventional greenhouses [3,4]. In such cases, the required plant temperature is achieved through the heating of the greenhouse air up to a temperature that is equal or slightly higher than the requisite one, thus creating a “whole” climate. Such conventional systems suffer from increased heat losses by convection and radiation through the cover, as well as through leaking by the inevitable construction flaws.

The aforementioned heat losses can be significantly reduced by direct plant heating with infrared (IR) radiation. In IR heating systems, the thermal energy is directly delivered to the plant canopy. As a result, temperature of the internal greenhouse air and cover remain at values that are closer to the external environment, thus reducing heat losses and lower energy consumption by 40–50% [5,6]. Concurrently, IR systems provide advantageous environmental conditions in the plant canopy through the creation of a “local” microclimate, which leads to an overall improvement of the final product quality [7]. In such conditions, the probability of moisture condensation on plants’ surface is significantly lower, the uniform quantitative and qualitative growth of plants is promoted, and plant pest and disease expansion are suppressed [8]. Finally, the IR systems present low thermal inertia by responding very fast to external temperature changes, as they only have to compensate for heat losses in the plant canopy.

Eggplants (*S. melongena* L.) are one of the four major crops in Greek greenhouses, along with tomatoes, peppers, and cucumbers, and, as such, were chosen for the current experiment. According to the literature data, eggplant is a rich source of phenolics, mainly anthocyanins and phenolic acids, and it is among the top ten vegetables with significant properties, such as antioxidant, antiradical, antiallergic, hypolipidemic, and anticancer [9,10,11]. Moreover, their relatively low height and small plant-canopy are suitable for cultivation within the two small experimental greenhouses.

The present study focuses on the effects of IR heating affects plant growth parameters, including the yield of the fruits, as well as the total phenolic content and the antioxidant profile of eggplants fruits’ extracts. This work is in continuation of a previous research study of our group dealing with the effects of IR heating and photovoltaic shading on pepper greenhouse cultivation [12]. Two identical small-scale experimental greenhouses were used, each one as the test bed of the two heating options that were considered, namely, forced hot air heating (conventional heating) and IR heating. Parallel operation of the two greenhouses allows for a comparison of the two heating systems under identical weather conditions and their effect to plant growing with plant growing parameters’ evaluation.

Greenhouse energy profile and plant growing parameters were examined. A step further, the effect of IR heating on the phenolic profile of eggplants was studied, measuring the in vitro antioxidant activity while using 2,2’-azino-bis-(3-ethylbenzothiazoline-6-sulfonic acid) radical (ABTS^+^) and Ferric Reducing/Antioxidant Power (FRAP) assays in relation to total phenolic content, as well as characterizing the (poly)phenolic Liquid Chromatography–Mass Spectrometry (LC–MS) profile of eggplant fruits from IR and conventionally heated greenhouses.

## 2. Materials and Methods

### 2.1. Chemicals, Standards and Solvents

All of the reagents and solvents that were used for the spectrophotometric assays and spectrometric (LC-MS) techniques were of High-Performance Liquid Chromatography (HPLC) grade and obtained from Mallinckrodt Chemical Works (St. Louis, MO, USA), Alfa Aesar GmbH & Co (Karlsruhe, Germany), Acros Organics (Belgium, WI, USA), and Sigma-Aldrich Chemie GmbH (Darmstadt, Germany).

### 2.2. Greenhouse Experimental Setup

**Figure 1:**
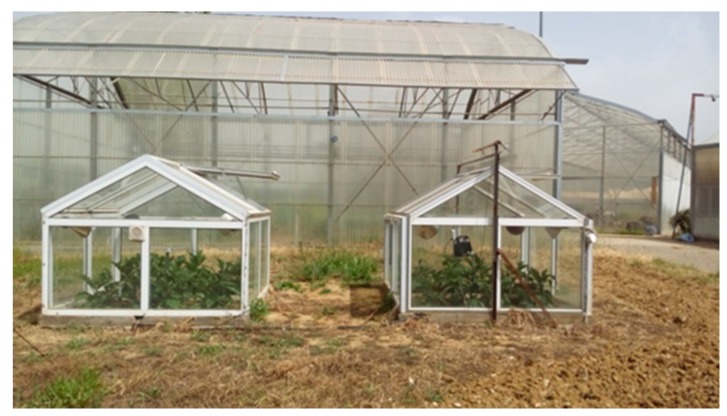
The experimental setup for the eggplant greenhouse cultivation.

The experimental design consisted of two identical small-scale greenhouses of total volume (V) 5.33 m^3^ each. The base area (Ap) of each greenhouse was 4.26 m^2^, the cover area (Ac) was 14.05 m^2^, and their dimensions were: width 2.13 m, length 2.00 m, eaves height 1.00 m, and total height up to the top 1.50 m. The glass thickness of the panels was 3 mm. Two different heating systems were used in the experiment. In the first greenhouse, where conventional heating was applied, hot air was forced by an air unit of two power levels (1 and 2 kW) that were equipped with a small fan that promoted air mixing. In the second greenhouse, IR heating was applied, which consisted of four lamps with blown bulb reflectors (1 kW total power, 50° beam angle) that were placed at the greenhouse corners and an elevation of 1 m above the plants (Figure 1). Both greenhouses had the same orientation and they were located side by side in the same field with a sufficient distance that excluded mutual interference.

Temperature was monitored both in the interior and the exterior of the greenhouses during the three (3) months operation period (February to April) with data loggers. In the interior, temperature was measured at several locations, whereas outdoor temperature was recorded on a meteorological station that was positioned close to the greenhouses. Temperature data were recorded every minute and 10-min. averages were stored with an analogue multiplexer relay. Overall day-time and night-time average values were computed for the time series, based on the interval between the steep radiation and temperature changes at sunrise and sunset. The Analyzer 4.5 Data logger Software (Scientific Enterprises Ltd, Moschato Atica, Greece) was used for the processing and statistical analysis of the experimental data.

Seeds of the *Solanum melongena* L. variety were used and sexual reproduction techniques were performed in a seedbed greenhouse. The seeds were planted at a depth of 3 cm and lightly watered at the same time in pots with pre-disinfected soil (previcur pesticide).

At a constant seedbed temperature of 25 °C, the seeds germinated after seven days and, after a total 40 days, the final seedlings were acquired ready for transplanting in the experimental greenhouse.

Daily moisture tests were performed during the 40 days of seedling production. Every 15 days (two times total) phosphorus fertilizers were applied and the atonik growth regulator was used to enhance rooting. On the day of planting the seedlings in the greenhouse, a spray with a copper based kocide fungicide was applied to prevent fungal diseases.

In each greenhouse, 16 young eggplants were planted on the soil, forming four rows of four plants each. The planting distances were 36 cm × 24 cm and plants were watered by a drip irrigation system, which supplied 2 L/h per drip. The irrigation dose during the experiment was 3.6 L per plant. Fertilizing of the plants was done with water-soluble fertilizers. During the cultivation, 120 g N, 280 g P, 160 g K, 12 g Mg, and 18 g Ca were administered on each plant. Prior to the installation of the plants, soil analysis data were considered to adjust the fertilization program. During the gradual ripening of the fruits, a total of four fruit batches were harvested and the full weight, dry weight, perimeter, and length of each fruit were measured.

Based on corresponding temperature recommendations, a night-time temperature of 16 °C was chosen for the timely growth of eggplant and the heating systems were set to turn on when the plants temperature (Tp) dropped below 15 °C and to turn off when Tp exceeded 17 °C.

### 2.3. Extraction Procedure

Eggplant fruits were freeze-dried. The preweighed samples of about 1 g were diluted in aqueous methanol (methanol: water 80:20, *v*/*v*) in a sample to solution ratio 1:20, for phenolic extraction. The extraction procedure was carried out in an orbital and linear motion shaker (Rotaterm, J.P. Selecta S.A., Barcelona, Spain) for 1 h at room temperature. The extracts were filtered and diluted to a final volume of 20 mL.

### 2.4. Spectrophotometric Studies

#### 2.4.1. Total Phenolic Content (TPC) Assay

The total phenolic content (TPC) of eggplant extracts was determined according to a modified micromethod of Folin–Ciocalteu’s colourimetric assay [13] and it was expressed as mg gallic acid equivalents (GAE) per 100 g of eggplant dry weight.

#### 2.4.2. Scavenging Activity on ABTS^+^ Radical

The antiradical activity of eggplant extracts was determined according to the method that was described by Lantzouraki et al. [14] and expressed as mg Trolox equivalents (TE) per 100 g of eggplant dry weight.

#### 2.4.3. Ferric Reducing/Antioxidant Power Assay (FRAP)

The FRAP assay of the eggplant extracts was carried out according to the method of Benzie and Strain [15], as modified by Lantzouraki et al. [16]. The antioxidant activity was expressed as mg FeSO_4_·7H_2_O per 100 g of eggplant dry weight.

### 2.5. Statistical Analysis

All data concerning production yield, growth parameters, and photometric assays were analyzed with One-Way ANOVA Post Hoc Tests. Pairwise multiple comparisons were conducted with the Tukey’s test. Probabilities lower than 0.05 were considered to be statistically significant (*p <* 0.05). All of the statistical calculations, including partial correlations, were performed with the SPSS package (IBM SPSS Statistics, version 19.0, Chicago, IL, USA) statistical software for Windows. The above parameters were measured in all of the fruits of 16 plants per greenhouse considered as replicates.

### 2.6. LC-MS Phenolic Profile

#### 2.6.1. Instrumentation

HPLC analysis was carried out while using an Agilent 1200 HPLC system installed with a G1379B micro vacuum degasser, a G1312A binary pump, a G1329 autosampler, and a G1316A oven (Agilent, Santa Clara, CA, USA). MS/MS experiments were conducted on a system that consisted of a 3200 Q TRAP triple-quadrupole linear ion trap mass spectrometer fitted with a Turbo V™ source and a Turbo Ion Spray probe (SCIEX, Framingham, MA, USA). Instrumental control, data acquisition, and processing were performed with Analyst Software program (version 1.4.2) (SCIEX, Framingham, MA, USA).

#### 2.6.2. Chromatographic Conditions

Chromatographic separation was performed on an Agilent Eclipse Plus C-18 reversed-phase column (50 mm × 2.1 mm inner diameter, 3.5 μm particle size) with a RRLC in-line filter kit (2.1 mm, 0.2 µm filter) (Agilent, USA). The binary solvent system consisted of solvent A (water-0.2% (*v*/*v*) formic acid) and solvent B (acetonitrile-0.1% (*v*/*v*) formic acid). The initial flow rate was 300 µL/min., with the column at room temperature and an injection volume of 5 µL. The gradient conditions of the mobile phase were linear 10%–20% B at 0.5 min. and linear 20%–30% B at 4 min. The flow rate was increased to 350 μL/min. and the gradient continued linear 30–50% B at 4.10 min., isocratic for 0.40 min., linear 50–65% B at 5.10 min., and linear 65%–100% B at 7 min., followed by isocratic elution for 1 min. The flow rate decreased back to 300 µL/min isocratic at 100% B for 1 more minute, linear 100%–10% B at 9.10 min., and finished with re-equilibration of the column, isocratic at 10% B from 9.10 to 15 min.

#### 2.6.3. Mass Spectrometry Analysis

The methanol extracts were diluted five times with methanol (0.1% formic acid, *v*/*v*) before filtration through a poly(tetrafluoroethylene) PTFE syringe filter (pore size 0.22 μm). The samples were injected in negative electrospray ionization (ESI) mode. Enhanced Mass Spectrum (EMS) survey scans and Information Dependent Acquisition (IDA)-triggered MS/MS scans (EPI-Enhanced Product Ion scans) were performed. The EMS survey scan was conducted at a mass range from 100 to 700 amu at a time of 0.1513 s. The scan rate was 4000 amu/s and the number of scans to sum was set to 3. The settings for the rest of the parameters were: curtain gas, 30 psi; collisionally activated dissociation (CAD) gas, medium; Temperature (TEM), 550 °C; gas 1 (GS1), 45 psi; gas 2 (GS2), 45 psi; ion spray (IS), −4500 V; declustering potential (DP), −30 V; entrance potential (EP), −7.50 V; collision energy (CE), −10 eV; and, collar 2 barrier (C2B), −300 V.

The IDA threshold was set to 100,000 counts. EPI scans were automatically performed for the two most intense peaks for each cycle, at masses ranging from 50 to 700 amu with a scan rate of 1000 amu/s. Linear Ion Trap (LIT) dynamic fill time was used. The number of scans to sum was set to 1 and the Q1 resolution was set to low. The rest of EPI scans settings were the following: curtain gas, temperature, gas 1 (GS1), gas 2 (GS2), ion spray (IS), declustering potential (DP), and entrance potential (EP) same values as for EMS survey scan; collisionally activated dissociation (CAD) gas, high; collision energy (CE), −30 eV; collision energy spread (CES), 10 V; collar 2 barrier (C2B), −450 V.

## 3. Results and Discussion

### 3.1. Temperature Results

**Figure 2:**
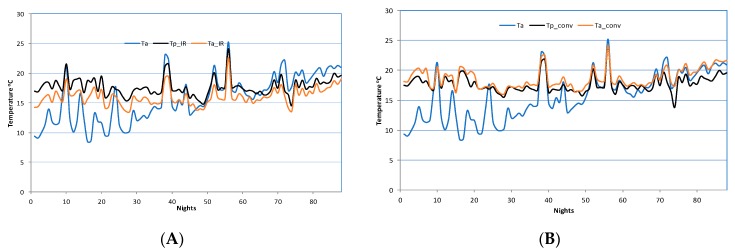
Variation of nightly ambient and inside greenhouses temperatures in (**A**) Infrared (IR) and (**B**) conventionally heated greenhouse. Ta: ambient temperature, Ta_IR & Ta_conv: inside temperature and Tp_IR & Tp_conv: plants temperature.

Figure 2A shows the variation of the nightly temperatures during the three months period for the IR heating greenhouse. The presented graphs are for the ambient temperature (Ta), the inside temperature (Ta_IR), and the plants temperature (Tp_IR) of the IR greenhouse. The diagram shows that the air temperature inside the greenhouse is lower than the plants temperature, creating a local environment on plant canopy in accordance with the IR radiation operation principles.

Figure 2B shows the variation of the nightly temperatures and, more specifically, the ambient temperature (Ta), the inside temperature (Ta_conv), and the plants temperature (Tp_conv) for the conventional heating greenhouse during a period of three months. The diagram shows that the air temperature inside the greenhouse is the same or slightly increased when compared to the target value for the plants, creating an isothermal environment throughout the greenhouse interior (whole climate).

### 3.2. Cultivation Results

**Figure 3:**
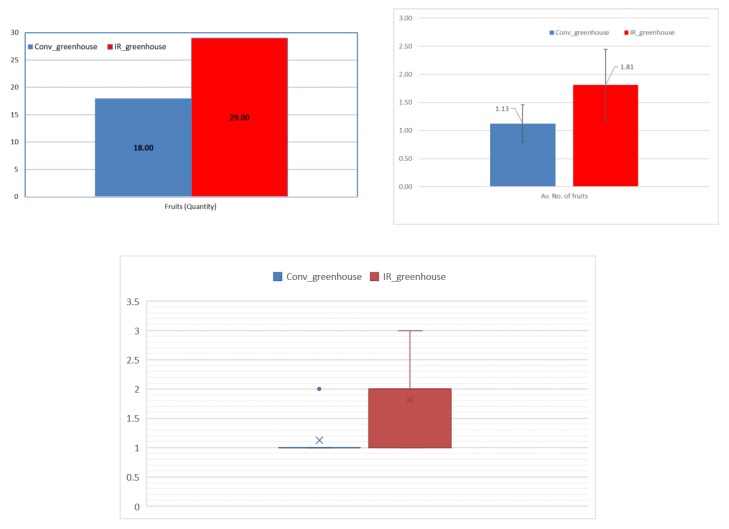
Production yield (upper left) absolute values; (upper right) mean values; and, (low center) median values of eggplant fruits, for each greenhouse.

Figure 3 presents differences between the two experimental sets for fruit quantity. Fruit production in absolute values was 18 fruits for the conventional and 29 for the infrared greenhouse. Although the differences in the average numbers of the overall fruit production are not statistically significant, an increase in the IR heating greenhouse against the conventional greenhouse is observed from the median values. This is of importance, since only two greenhouses were included for the study, but 16 plants were used in each greenhouse.

**Figure 4:**
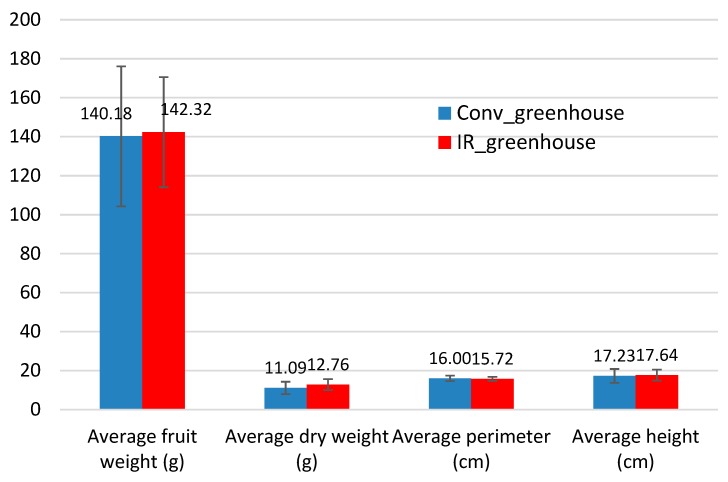
Average values of eggplant fruit growth indicators.

Figure 4 shows the average values regarding the number of fruits per plant, the fruit weight, and dry fruit weight, as well as the average of perimeter and height. These values do not show any statistically significant differences between the two greenhouses and uniform fruits were produced for both cases.

### 3.3. Total Phenolic Content (TPC) Antiradical and Antioxidant Activity of Eggplant’ Fruits

**Figure 5:**
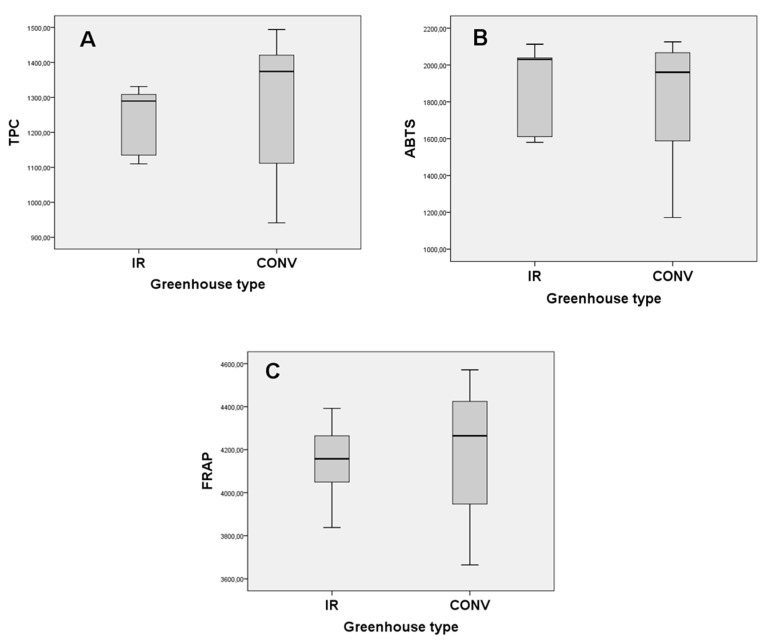
Boxplot diagrams of total phenolic content (**A**), antiradical (**B**), and antioxidant (**C**) activity of eggplant’ fruits from IR and conventionally heated greenhouses.

Total phenolic content (TPC), antiradical and antioxidant activity of the eggplant fruits are given as boxplots (A–C), respectively, as in Figure 5. Total phenolic content of eggplant samples from the greenhouse conventionally heated and with IR ranged from 941.63 to 1493.91 mg GAE/100 g DW and from 1109.78 to 1330.56 mg GAE/100 g DW, respectively. The TPC results of the studied eggplant samples were similar to those that were reported by Hanson et al. [17], for 35 eggplant species from different origins (740–1430 mg of chlorogenic acid equivalents /100 g DW), higher than those that were reported by Raigón et al. [18], for 31 eggplant cultivars (562–965 mg of chlorogenic acid equivalents /100 g DW) and lower than those that were reported by Niño-Medina et al. [19], for five commercial eggplant types from Mexico (1350–2049 mg of gallic acid equivalents per 100 g DW). It is important to mention that many factors may affect TPC, such as the harvesting season, the temperature, the intensity and type of light, the eggplant species, the eggplant type (wild or cultivated), the eggplant size, agronomic and genetic factors, etc. [9,20,21].

The antiradical activity, as determined by the ABTS assay, of eggplant fruits from the conventional and IR heating greenhouses ranged from 1171.67 to 2125.64 mg Trolox equivalents (TE) per 100 g DW and from 1580.27 to 2113.01 mg TE per 100 g DW, respectively. Moreover, and according to the FRAP assay, the antioxidant activity of eggplant extracts from the conventional and IR heating greenhouses ranged from 3694.88 to 4384.18 mg FeSO_4_ × 7H_2_O per 100 g DW and from 3837.84 to 4391.83 mg FeSO_4_ × 7H_2_O per 100 g DW, respectively.

Very strong positive correlations were found, between the TPC and FRAP assays (*r*^2^ = 0.917, *p <* 0.01), between the TPC and the ABTS assay (*r*^2^ = 0.920, *p <* 0.01), as well as among the ABTS and FRAP assays (*r*^2^ = 0.841, *p <* 0.01). In full agreement with our findings, Chumyam et al. [22], Okmen et al. [23], and Plazas et al. [24] reported high positive correlation among TPC and antioxidant activity in eggplant.

The homogeneity of variances of total phenolic content and antioxidant activity presented statistically significant differences (*p =* 0.0129 and *p =* 0.0344, respectively) between the eggplant fruits from conventional heating greenhouse and those from IR heating greenhouse (Figure 5), whereas the antiradical activity presented no statistically significant difference (*p =* 0.1061). Therefore, the results exhibited that the IR heating system has achieved eggplant production with greater uniformity in terms of phenolic profile than conventional heating, which is highly desirable for both statistical and commercial purposes.

### 3.4. Characterization of Phenolic Profile of Eggplant Fruits’ Extracts by LC-MS

A qualitative LC-MS^2^ approach was implemented to identify the (poly)phenolic profiles of eggplant fruits from IR and conventionally heated greenhouses. Table 1 lists the identified compounds in eggplant fruits’ extracts, along with their retention times (Rt), their negative [M−H]^−^ ions, and their MS^2^ fragmentation data. For the identification of phenolic compounds, the parent ions (*m*/*z*) that were obtained from the MS^1^ and fragments from the MS^2^ spectra were comparatively studied with those from respective literature data. According to Table 1, thirty and twenty-five phenolic compounds were identified in the eggplant fruits from IR and conventional heating greenhouses, respectively.

Among the most interesting findings was the putative identification in the eggplant samples of brevifolin carboxylic acid, acacetin, and cosmosiin, irrespective of the type of greenhouse heating. According to the literature, brevifolin carboxylic acid has been shown to inhibit hepatitis B virus replication and tumor growth [25], whereas acacetin has been found to be a potential therapeutic for the prevention or treatment of Alzheimer’s disease [26]. Furthermore, cosmosiin has demonstrated significant liver protective properties and it has been found to reduce hepatocyte damage [27].

Dihydrokaempferol, p-hydroxy benzoic acid hexoside, epigallocatechin or gallocatechin, trihydroxy-octadecadienoic acid, ferulic acid-hexoside, 3-acetyl-5-caffeoylquinic acid, 1-*O*-caffeoyl-2-*O*-glucosylglycerol, lariciresinol glucopyranoside, and dimeric procyanidin were only identified in the eggplant fruits from IR heated greenhouses. Moreover, dihydrokaempferol-hexoside, ellagic acid-hexoside, *N*1,*N*4-dicaffeoyl spermidine, and rutin were only identified in the eggplant fruits from conventionally heated greenhouses. Given the fact that changing environmental factors, such as light quality, electromagnetic radiation type, and temperature, significantly influence the secondary metabolism of plants, it seems that the heating process has significantly affected the profile of phenolic compounds that are produced by growing eggplants. Interestingly, infrared heating promoted the lariciresinol glucopyranoside biosynthesis, a bioactive lignin glycoside with noteworthy antiviral properties [28], in cultivated eggplants. Moreover, infrared heating appears to have favoured the flavan-3-ols’ production, e.g., dimeric procyanidin and epigallocatechin or gallocatechin, which are known to exhibit a wide range of biological and pharmacological properties, such as anti-inflammatory, antimicrobial, and antioxidant [29]. Contradictory, conventionally heating seems to stimulate the biosynthesis of *N*1,*N*4-dicaffeoyl spermidine and rutin. Based on the above results, it appears that infrared heating might affect the increasing biosynthesis of some metabolites instead of others.

Moreover, from the above-mentioned phenolic compounds, the 5-o-caffeoylshikimic acid, chlorogenic acid, 3-acetyl-5-caffeoylquinic acid, isochlorogenic acid, feruloylquinic acid, and *N*1,*N*4 -Dicaffeoyl spermidine have been previously identified in other eggplant cultivars [21,30].

Finally, flavonol (quercetin, kaempferol, and myricetin) derivatives were identified in eggplant fruits, irrespective the heating process, in accordance to the findings that were reported by García-Salas et al. [21], for diverse cultivars of eggplant grown in different seasons and by Singh et al. [30], for eggplant skins that were grown in organic and conventional environments.

## 4. Conclusions

The present study focuses on the effects of IR heating in greenhouse installations on eggplant cultivations. Specific plant parameters were studied, including fruit growth and yield, as well as alterations in the phenolic profile of eggplants as a result of IR radiation.

To implement the study, two identical small-scale experimental greenhouses employing IR and conventional heating systems, respectively, were used for eggplants production. The IR heating established a smooth temperature distribution at the canopy, while inside greenhouse the air was maintained at lower values than in the conventionally heated greenhouse. Eggplant fruit growth was similar in both greenhouses, while the production yield was significantly increased in the IR heating greenhouse.

Total phenolic content, antioxidant, and antiradical activity were measured showing a higher value distribution in eggplant fruits from the conventional compared to the IR heating greenhouse. This results in greater uniformity production in terms of phytochemical profile in the IR heating greenhouse, which could have a positive impact in commercial applications.

The qualitative LC-MS phytochemical profile of eggplant fruits from both greenhouses revealed the existence of numerous bioactive compounds, some of which were only characteristic of the eggplant fruits from IR heated greenhouses. Overall, it was revealed that IR radiation influenced the secondary metabolism of eggplants through the enhancement of the biosynthesis of specific compounds over others.

## Figures and Tables

**Table 1:** (poly)Phenolic compounds in eggplant’ fruits extracts from IR and conventionally heated greenhouses.

No	RT (min)	Compound (Tentative Identification)	[M−H]^−^	MS^2^ [M−H]^−^	IR Heated Greenhouse	Conventionally Heated Greenhouse	References
1	0.484	Caffeic acid-hexoside	341	179, 161, 135	+	+	[31,32]
2	0.542	Quinic acid	191	173, 129, 111, 93, 85	+	+	[33,34]
3	0.803	l-malic acid	133	115, 87, 71	+	+	[35]
4	0.987	Chlorogenic acid	353	191, 179, 161, 135, 93	+	+	[36]
5	1.057	Myricetin-3-*O*-glucoside	479	354, 317, 191, 179	+	+	[14]
6	1.512	Isochlorogenic acid	515	408, 395, 353, 191, 179	+	+	[37]
7	1.691	*p*-Hydroxybenzoic acid	137	109, 93	+	+	[38,39]
8	2.035	Catechin hexoside	451	289, 245, 179, 167	+	+	[40]
9	3.196	Dihydroxycinnamoyl amide	470	334, 309, 191, 179, 135	+	+	[30]
10	3.556	Feruloylquinic acid	367	279, 193, 191, 173	+	+	[41]
11	3.607	*N*1,*N*4-Dicaffeoyl spermidine	468	332, 306, 291, 276, 161		+	[30,40]
12	3.662	5-*O*-caffeoylshikimic acid	335	179, 173, 161, 135, 93	+	+	[42]
13	3.709	Ellagic acid-hexoside	463	301, 300, 257		+	[14]
14	3.942	3-acetyl-5-caffeoylquinic acid	395	353, 233, 191, 179	+		[30]
15	3.957	Rutin (Quercetin *O*-rhamnosyl-*O*-hexoside)	609	463, 343, 301, 285		+	[43]
16	3.999	Lariciresinol glucopyranoside	521	359, 341, 329, 187, 160	+		[44]
17	4.021	6-Prenyl-naringenin	340	323	+	+	[45]
18	4.167	Dihydrokaempferol-hexoside	449	431, 287		+	[14]
19	4.533	Gallic acid monohydrate	187	169, 125, 97	+	+	[38,40]
20	5.584	Caffeic acid	179	135, 91	+	+	[14,38,39]
21	5.682	Dimeric procyanidin	577	451, 425, 289, 202	+		[14,39]
22	6.320	Epigallocatechin or Gallocatechin	305	261, 219, 191, 179, 125	+		[39]
23	6.497	Brevifolin carboxylic acid	291	247, 203	+	+	[14,35]
24	6.598	Galloyl hexoside	331	211, 169, 151, 125	+	+	[46]
25	6.626	Vanillic acid-4-*O*-hexoside	329	293, 284, 269, 209, 181, 167	+	+	[14,34]
26	6.741	Dihydrokaempferol	287	269, 259, 243, 201, 125	+		[40]
27	6.843	Cosmosiin	431	271, 225, 153, 125	+	+	[37]
28	6.984	Trihydroxy-octadecadienoic acid isomer	327	206	+		[42]
29	7.151	1-*O*-Caffeoyl-2-*O*-glucosylglycerol	415	253, 179, 161, 135	+		[40]
30	8.029	Quercetin 3,7-di-*O*-*α*-l-rhamnopyranoside	593	498, 432, 414, 316, 278, 241, 224, 153	+	+	[34]
31	8.447	p-hydroxy benzoic acid hexoside	299	255, 162	+		[34]
32	8.799	Naringenin	271	254, 226, 177, 151	+	+	[39,47]
33	9.540	Ferulic acid-hexoside	355	265, 217, 193, 175	+		[14,43]
34	10.365	Acacetin	283	270, 242	+	+	[37]
